# Development, structure and mechanics of a synthetic *E. coli* outer membrane model†

**DOI:** 10.1039/d0na00977f

**Published:** 2020-12-16

**Authors:** Bálint Kiss, Tamás Bozó, Dorottya Mudra, Hedvig Tordai, Levente Herényi, Miklós Kellermayer

**Affiliations:** Department of Biophysics and Radiation Biology, Semmelweis University Budapest Hungary kellermayer.miklos@med.semmelweis-univ.hu

## Abstract

The outer membrane (OM) of Gram-negative bacteria is a complex asymmetric bilayer containing lipids, lipopolysaccharides (LPS) and proteins. While it is a mechanical and chemical barrier, it is also the primary surface of bacterial recognition processes that involve infection by and of the bacterium. Uncovering the mechanisms of these biological functions has been hampered by the lack of suitable model systems. Here we report the step-by-step assembly of a synthetic OM model from its fundamental components. To enable the efficient formation of a supported lipid bilayer at room temperature, dimyristoyl-phosphocholine (DMPC) was used as the lipid component to which we progressively added LPS and OM proteins. The assembled system enabled us to explore the contribution of the molecular components to the topographical structure and stability of the OM. We found that LPS prefers solid-state membrane regions and forms stable vesicles in the presence of divalent cations. LPS can gradually separate from DMPC membranes to form independent vesicles, pointing at the dynamic nature of the lipid-LPS system. The addition of OM proteins from *E. coli* and saturating levels of LPS to DMPC liposomes resulted in a thicker and more stable bilayer the surface of which displayed a nanoscale texture formed of parallel, curved, long (>500 nm) stripes spaced apart with a 15 nm periodicity. The synthetic membrane may facilitate the investigation of binding and recognition processes on the surface of Gram-negative bacteria.

## Abstract 2

The outer membrane (OM) of Gram-negative bacteria is a complex asymmetric bilayer containing lipids, lipopolysaccharides (LPS) and proteins.

## Graphical abstract

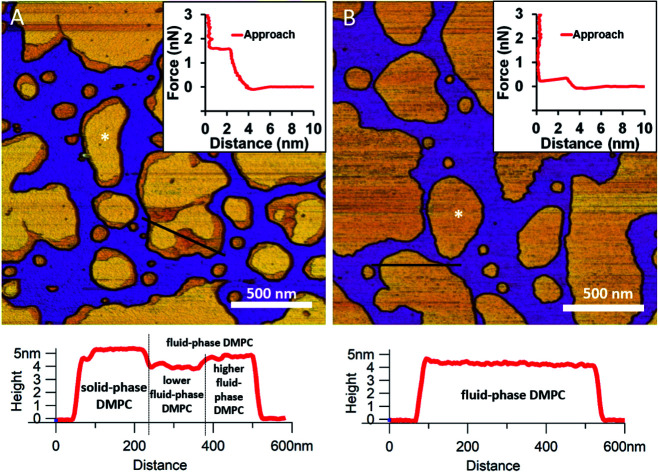

## Introduction

The outer membrane (OM) of *E. coli* is a multi-functional asymmetrical membrane system responsible for transporting nutrients into the cytoplasm while isolating it from harmful agents in the environment. Its fundamental components are lipids, lipopolysaccharides (LPS) and various types of proteins. Infection by bacteriophages such as T7,^1–3^ is thought to be intimately linked to the structure and properties of the OM. In spite of the importance of the OM in bacterial biology and pathology, our knowledge about its structure and biophysical characteristics is still limited. About 75% of the OM surface is made up of lipopolysaccharides (LPS) which are highly heterogeneous, negatively charged molecules. The remaining area is occupied by proteins.^4,5^ In rough type LPS only the core region is present, whereas in smooth type variants the oligosaccharide chain is much longer, resulting in a several-nanometer-thick layer. Subgroups of rough type LPS are Re, Rd, Rc, Rb and Ra in growing oligosaccharide chain length order.^6^ LPS is responsible for inducing various biological reactions as it initiates a strong immune and toxic response in the host.^7^ Furthermore, it has recently been shown that LPS might play a role in the initial phase of the host-recognition process of bacteriophages.^3^ Tong *et al.* found that Rd-type LPS-containing bilayers can be efficiently formed on positively charged surfaces if the samples were heated to 60 °C and kept at this temperature for several hours.^8^ Kaufmann *et al.* observed that the LPS membrane can be ruptured, with atomic force microscopy (AFM), in a single breaking-through event similarly to a lipid bilayer, suggesting that their supramolecular structures are similar.^9^ The native OM of live bacteria is quite challenging and difficult to image *in situ* due to its three-dimensional shape and dynamic behaviour. In a successful attempt, bacterial cells were allowed to adhere to lithographically patterned or gelatin-coated surfaces, which resulted in sufficiently restricted movement so that they could be imaged in growth media.^10,11^ Drying *E. coli*-containing samples on a supporting surface has also been employed to study the effect of divalent cations on the cell wall structure.^12^ Although drying aids cellular immobilization, it introduces unwanted artefacts. OM proteins of *E. coli* have been reconstituted into lipid bilayers,^13,14^ but these lacked the LPS component. In a novel approach, OM models can also be generated from outer membrane vesicles, but controlling the amounts of OM proteins and LPS may pose a challenge.^15^

In the present work we developed a synthetic bacterial membrane model by employing step-by-step construction from the main constituents. The two leaflets of the natural OM are very different in their composition, whereas the models presented here are all symmetrical, mimicking the outer leaflet of the OM on both sides. Lipids with phosphocholine headgroups have been used successfully to create OM models.^16^ The addition of LPS to phospholipid membranes resulted in a rough surface texture and an additional penetrable layer. Incorporation of OM proteins extracted from *E. coli* further increased membrane thickness and stability, indicating that the bacterial outer membrane is mechanically reinforced by LPS and proteins.

## Experimental

### Materials

1,2-dimyristoyl-*sn*-glycero-3-phosphocholine (DMPC) was from Avanti Polar Lipids Inc. (Alabaster, AL). Lipopolysaccharides (LPS, rough strains) from *Escherichia coli* EH100 (Ra mutant), Whatman Nuclepore membrane filters (track-etched polycarbonate membranes, pore diameter 100 nm), octyl glucopyranoside (OG), sarkosyl, poly-l-lysine (PLL) (150 000–300 000 g mol^−1^), lysozyme, sodium azide, calcium, Trizma base and 1 M magnesium chloride solutions were from Sigma-Aldrich. DNAse I was from Thermo Fisher Scientific. Argon 5.0 and nitrogen 5.0 gases were from Linde Gáz Magyarország Zrt (Budapest, Hungary). Water was purified with a Milli-Q Integral 3 Water Production Unit (Merck Millipore, Billerica, MA). Round mica sheets were from Ted Pella, Inc. (Redding, CA). Detergent adsorbent SM-2 Bio Beads were from Bio-Rad.

### Methods

#### Preparation of DMPC and LPS-DMPC vesicles

Small unilamellar vesicles (SUVs) were prepared by the extrusion method.^17^ Briefly, 10 mg ml^−1^ DMPC/ethanol stock solution was added to a clean glass tube, the solvent was evaporated in Ar gas stream then in vacuum. Desired amounts of LPS dissolved in purified water was added, and the sample was further diluted with TRIS buffer (20 mM TRIS-HCl, 140 mM NaCl, pH 7.4). TRIS buffer was used throughout the experiments unless stated otherwise. The final lipid sample, at a total lipid concentration (which included LPS) of 1 mg ml^−1^, was extruded through a polycarbonate membrane using an Avanti Mini Extruder (Avanti Polar Lipids) at 40 °C. The amounts of DMPC and LPS stock solutions added depended on the targeted final LPS concentration (*e.g.*, 0.5 mol mol^−1^%) of the sample. LPS content greater than 10% was not achievable due to LPS solubility limitations. These high amounts were well above the ratio limitations that could be completely embedded into DMPC bilayers, thus images of LPS-DMPC samples shown are those with LPS ratios of 0.5%. It should be noted that the purchased EH100 LPS contained traces of proteins as noted by the manufacturer (Fig. S1†). The resulting liposomes were stored at room temperature in TRIS buffer. The procedure to prepare DMPC and LPS-DMPC vesicles was repeated more than twenty times with the same experimental outcome.

#### Preparation of DMPC + LPS SLBs

DMPC supported lipid bilayers (SLBs) were prepared as described in the AFM imaging section. To obtain LPS-containing SLB (“LPS-DMPC”), 100 μl of 20 μg ml^−1^ LPS solution was incubated on top of the SLBs for 15 minutes. Then the sample was washed five times with TRIS buffer containing 10 mM CaCl_2_ or MgCl_2_ to remove unbound free LPS and stabilize LPS-DMPC. Alternatively, a detergent-mediated reconstitution method was used which resulted in larger LPS incorporation ratios (see below).

#### Preparation of bacterial OM extract

Bacterial cell wall extraction was carried out as described by Filip *et al.*, with modifications.^18^*E. coli* (ATCC 11303) bacterial cell cultures were grown in LB broth until an OD_550_ of 1.0 was reached, then the cells were collected by centrifugation. The bacterial cell wall was disrupted with 0.2 mg ml^−1^ lysozyme, and 1 μg ml^−1^ DNAse was added to digest bacterial DNA. Cells were lysed by sonication (0.5 s ON–1.0 s OFF cycles, 75 W) on ice for 15 min. Ultracentrifugation was used to collect the membrane fraction (100 000×*g*, 1 h, 4 °C). To solubilize internal membranes, the pellet was resuspended in 2 w/v% sarkosyl buffer (20 mM TRIS-HCl, pH 8) and incubated for 45 min. The OM fraction was collected with ultracentrifugation (100 000×*g*, 1 h, 4 °C). The pellet was resuspended in TRIS buffer, aliquoted and flash frozen in liquid nitrogen, then stored at −80 °C until further use. The frozen aliquots were used in OM-DMPC preparation. OM extracts were analysed by SDS-PAGE (10% acrylamide) (Fig. S1†). Due to the implemented extraction method OM extracts contained high amounts of proteins as well as LPS. The procedure to prepare OM extracts was repeated more than five times with the same experimental outcome.

#### Reconstitution of OM proteins into DMPC liposomes

Reconstitution was based on a method used before by Plançon *et al.*^19^ Briefly, a concentrated OM aliquot was diluted in 40 mM OG solution to solubilize the membrane proteins, mixed with DMPC SUVs prepared in advance (3 mg ml^−1^ final concentration), and incubated for 15 min. OG was chosen as the detergent based on numerous successful OM protein reconstitution experiments.^20^ To reconstitute the proteins into DMPC liposomes, the detergent was slowly removed to facilitate the formation of proteoliposomes (OM-DMPC). Detergent-adsorbing SM-2 Bio-Beads were used at a starting concentration of 50 mg ml^−1^ for 1 h. Subsequently, the same amount of beads was added, and the sample was further incubated at room temperature for 1 h to completely remove the detergent.^21^ The beads were removed by filtration through Pierce Spin Columns (Thermo Scientific). The procedure to prepare OM-DMPC samples was repeated more than ten times with the same experimental outcome.

#### Preparation of high-LPS-content DMPC vesicles

To increase the LPS content of DMPC vesicles, we used detergent-mediated reconstitution to incorporate pure LPS (10%, meaning 20 times the ratio achievable with extrusion) into DMPC bilayers. The procedure to prepare high-LPS-content vesicles was repeated more than five times with the same experimental outcome.

#### Dynamic light scattering (DLS)

Size distribution of liposomes was measured by using a custom-built DLS equipment comprising a goniometer system (ALV GmbH, Langen, Germany), a diode-pumped solid-state (DPSS) laser light source (Melles Griot 58-BLS-301, 457 nm, 150 mW) and a light detector (Hamamatsu H7155 PMT module). The scattering angle was 90°. For DLS measurements, 50 μl of the sample was diluted to a final volume of 300 μl. The contribution of different particle species to the autocorrelation function was determined by the maximum entropy method (MEM),^22^ and, with the assumption of spherical shape, the particle size distribution was calculated.^23^ Measurements were carried out at room temperature.

#### AFM imaging

A SUV aliquot (DMPC or LPS-DMPC, 1 mg ml^−1^) was diluted with TRIS buffer containing 10 mM CaCl_2_ (except where stated otherwise) to achieve a final volume and concentration of 100 μl and 20 μg ml^−1^ or 30 μg ml^−1^, respectively. The sample was pipetted onto a freshly cleaved mica surface and incubated to allow for SLB formation. After 15 min the sample was washed five times with 100 μl of 10 mM CaCl_2_ TRIS buffer. Non-contact imaging was carried out in the washing solution by using an Asylum Research Cypher ES instrument (Asylum Research, Santa Barbara, CA) with a silicon nitride cantilever (BL-AC40TS-C2, Olympus, Japan) at various temperatures (held constant with 0.1 °C accuracy). The cantilevers had typical spring constants of 90 pN nm^−1^ and were oscillated near their resonance frequencies by using blueDrive technology (photothermal excitation). Similar steps were used to image LPS vesicles at a loading concentration of 20 μg ml^−1^. For imaging OM-DMPC, samples were applied onto poly-l-lysine (PLL)-coated surfaces. PLL-coated substrate surface was prepared by pipetting 100 μl of PLL (0.1% w/v) onto freshly cleaved mica, followed by incubation for 20 min, repeated rinsing with purified water, and drying with a stream of high-purity nitrogen gas. AFM images were analysed with the built-in functions of the AFM driving software (IgorPro, WaveMetrics, Inc., Lake Oswego, OR). During temperature-dependent experiments the rate of temperature change was 1 °C s^−1^. After reaching the set temperature, the sample was always allowed to equilibrate for 15 minutes before imaging. The figures show height-(violet–orange–yellow color coded) and amplitude-contrast (brown-scale color coded) images. Post processing and scanning-artifact removal was executed with the AFM driving software. Surface roughness was calculated from at least three independent 2 × 2 μm images per sample.

#### Force spectroscopy

Force spectra were recorded at pre-selected points of the AFM image. The cantilever was moved perpendicular to the surface with a constant velocity of 0.5 μm s^−1^. The average acquisition time of a single force was three seconds (approach and retraction). During the acquisition of a single force spectrum the sample point was pressed with gradually increasing force until the typical pre-set level of 10 nN was reached. Median-filtered force–distance curves were analysed with a custom-written algorithm to find membrane-rupture steps with size and force greater than 0.1 nm and 0.1 nN, respectively. 400 force curves were used for analysis of DMPC (20 °C and 30 °C), LPS-DMPC and OM-DMPC samples.

## Results and discussion

### Model membranes prepared by extrusion

In this work we generated, by progressively mixing lipid, LPS and protein components, a synthetic membrane system resembling the composition and properties of the outer membrane of Gram-negative bacteria.

#### DMPC forms bilayers on mica surface

**Fig. 1:**
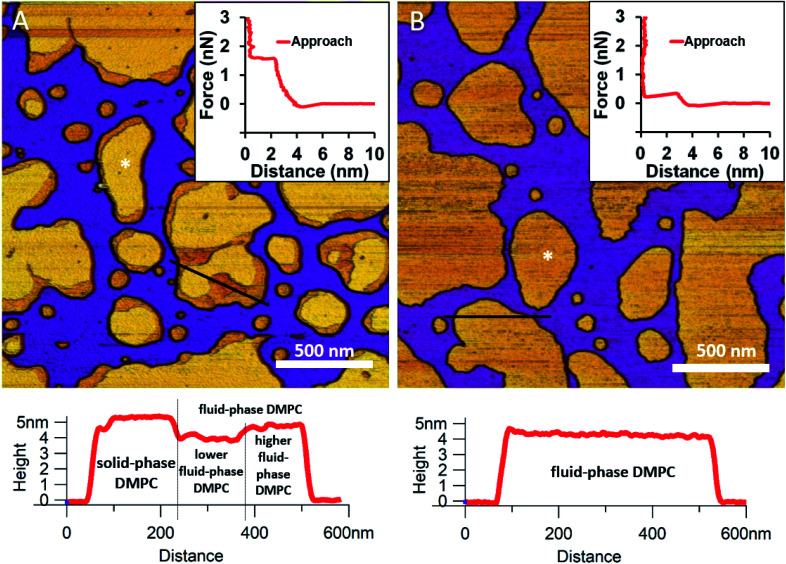
Height-contrast AFM images of DMPC SLBs: DMPC SLBs imaged at 20 °C (A). DMPC SLB heated to 30 °C (B). Insets: force–distance curves recorded while rupturing DMPC at the locations marked by the white star on each image. Lower panels show the height-distance functions along the black lines indicated on the images.

We began by forming a supported lipid bilayer (SLB) from liposomes. Fig. 1A shows the height-contrast AFM image of SLBs formed by incubating DMPC liposomes on mica at room temperature. In the presence of 10 mM Ca^2+^, the liposomes adhered to the surface and formed SLB patches. DMPC is often used as a constituent in liposome preparations due to its low main transition temperature (24 °C), which allows for liposome formation and surface adhesion even at room temperature.^24^ As the topographical height profile of the SLB shows, two distinct smooth-surface lipid domains formed with average topographical heights of 4 and 5 nm (Fig. 1A lower panel). The inner regions of the membrane patches are higher, whereas the rim of patches is lower. Although at this temperature (near the main transition temperature, 24 °C) DMPC was reported to form a homogeneous solid phase,^25^ the presence of structurally distinct regions indicates that two independent phases are present. The low (*i.e.*, thin) and high (*i.e.*, thick) regions likely correspond to the fluid and solid phases, respectively. Accordingly, the two phases coexist in the same SLB patch, but the solid phase dominates (65% solid, 35% fluid phase according to surface area). To test this idea, samples were heated to 30 °C which exceeds the transition temperature, at which DMPC is expected to form homogeneous fluid-phase SLBs. As Fig. 1B shows, SLBs indeed became homogeneous with a uniform height of 4 nm (100% fluid phase according to surface area), pointing at a complete solid-to-fluid main phase transition. Interestingly, the lower regions of DMPC can be further divided into two distinct phases according to height (Fig. 1A lower panel). This phenomenon may be explained by vertical phase separation, in which one leaflet of the bilayer is in the fluid while the other is in the solid phase. Typical force curves show that the mechanical rupture of DMPC bilayers occurs in a single step regardless of temperature (Fig. 1 insets). By contrast, rupturing DMPC at a higher temperature is much easier (mean rupture force 0.34 ± 0.08 nN at 30 °C compared with 1.3 ± 0.3 nN at 20 °C). The lower rupture forces of fluid-phase DMPC SLBs (30 °C) can be attributed either to weaker interaction between individual lipid molecules, or to a decrease in SLB density caused by spreading at higher temperatures.

#### LPS forms stable vesicular structures

**Fig. 2:**
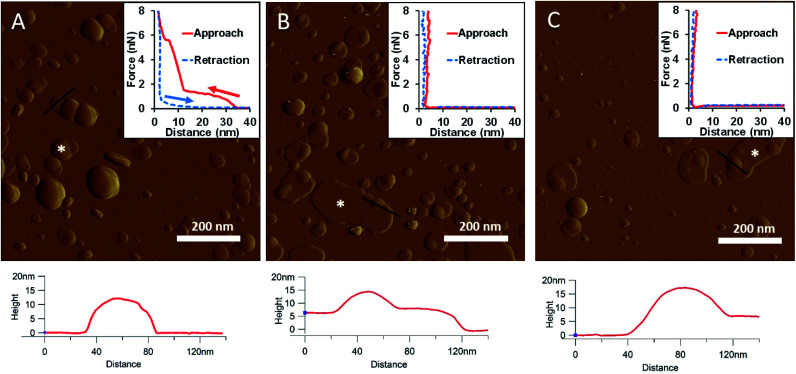
Amplitude-contrast AFM image of LPS vesicles in the presence of 10 mM Ca^2+^; at 20 °C (A). The sample was warmed to 50 °C (B); and then cooled back to 20 °C (C). Insets: force–distance curves recorded while pressing LPS at the locations marked by the white star on each image. On the inset of A arrows indicate the direction of tip movement. Lower panels show height profiles taken along the black lines.

**Fig. 3:**
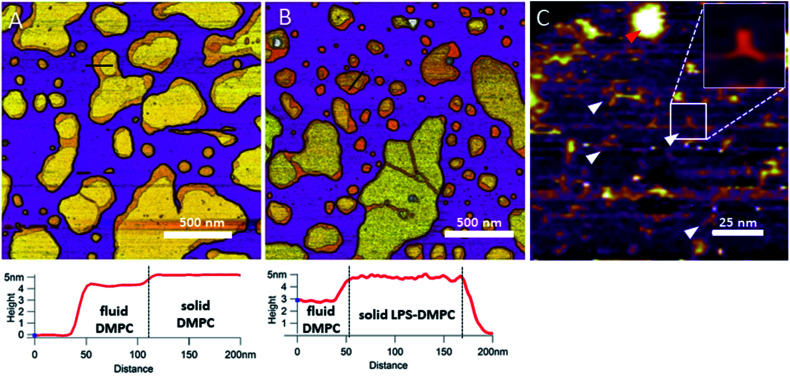
Height-contrast AFM image of DMPC SLB (A); LPS-DMPC SLB (B); and an LPS-rich region of LPS-DMPC SLB at high resolution (C); on mica at 20 °C in the presence of 10 mM Ca^2+^. Lower panels show the height-distance functions along the black lines shown in the images. In C the putative branching LPS oligosaccharide chains are marked with white arrowheads; one of them is enlarged in the top right inset. Red arrowhead indicates an LPS bump.

**Fig. 4:**
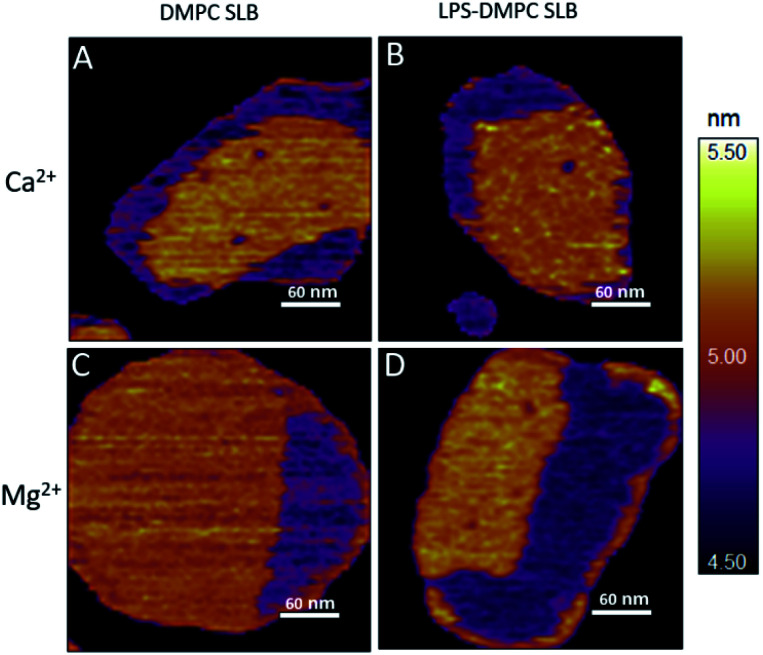
Height-contrast AFM image of DMPC SLB (A and C); LPS-DMPC SLB (B and D); on mica at 20 °C in the presence of 10 mM Ca^2+^ (A and B); in the presence of 10 mM Mg^2+^ (C and D) respectively. Orange and purple indicate solid and fluid states, respectively.

**Fig. 5:**
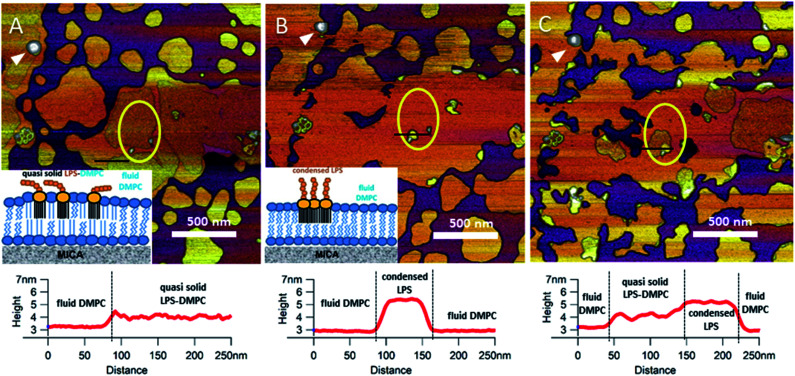
Height-contrast AFM image of the same region of LPS-DMPC SLB patches in the presence of 10 mM Ca^2+^. At 20 °C (A); warmed to 30 °C (B); and then cooled to 20 °C (C). Lower panels show the height-distance functions along the black lines shown in the images. LPS/protein-bumps marked with white arrowhead. Yellow ellipse shows a region of interest (ROI) for better understanding of possible processes. Insets show a simplified model of LPS interactions with solid-(blue lipids with straight fatty acid chain) and fluid-phase DMPC (sawtooth-shaped fatty acid chains).

**Fig. 6:**
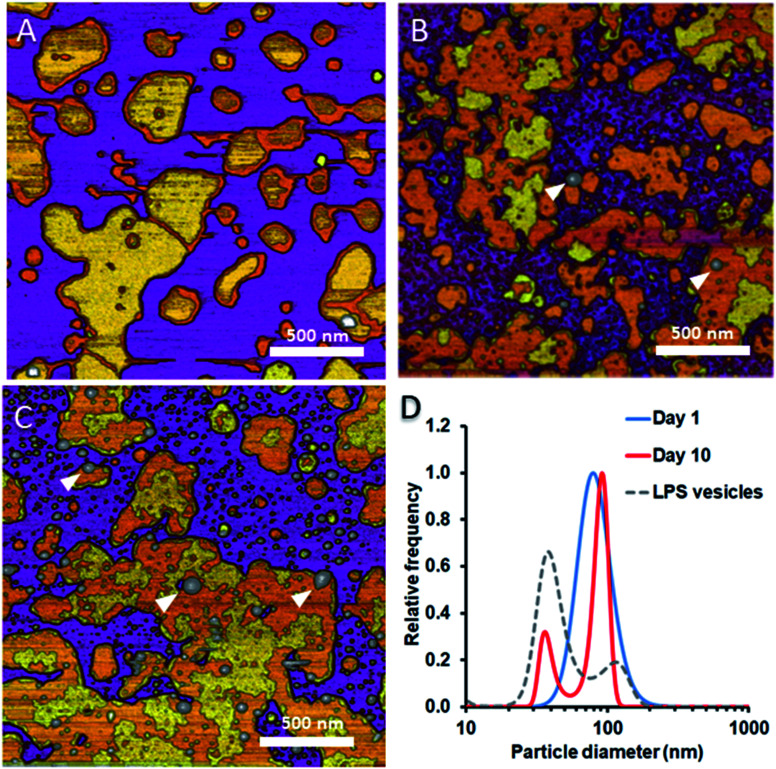
Height-contrast AFM images of LPS-DMPC SLB recorded as a function of time across a time span of 10 days. 0.5% LPS-DMPC patches formed by a freshly prepared (A); a 5 day-old (B); and a 10 day-old sample (C) on mica at 20 °C in the presence of 10 mM Ca^2+^. LPS separates from LPS-DMPC membranes and forms vesicles marked by white arrowheads in B and C. Size distribution of LPS solution and LPS-DMPC liposomes on day 1 and day 10 measured by DLS (D). Images were recorded at 20 °C.

**Fig. 7:**
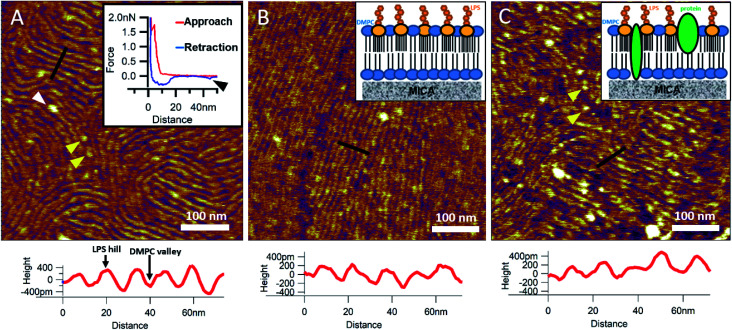
SLBs formed by liposomes created with detergent mediated reconstitution. OM-DMPC SLB surface texture at 15 °C (A). Inset shows the occasional protein unfolding (black arrowhead) recorded by FS on OM-DMPC membranes (see expanded view in Fig. S11†). White arrowhead shows LPS-protein aggregate. Imaging at 15 °C stabilizes the sample, thus better resolution can be achieved, however no structural rearrangement was observed. Surface of high-LPS-content DMPC bilayers prepared with detergents have a 2D crystalline wavelike pattern (B) similar to OM-DMPC samples (C). Both images were recorded at 20 °C. Insets show a simplified model of the SLBs. Yellow arrowheads show single proteins, similarly to the ones in the inset of A. Lower panels show height profiles taken along the black lines.

To generate a synthetic bacterial outer membrane that resembles the native one as close as possible, we incorporated LPS into the DMPC membrane. To uncover the effects LPS has on membrane structure and mechanics, we compared the properties of LPS vesicles with those of LPS-complemented DMPC membranes (Fig. 2–7). Similarly to DMPC liposomes, LPS needs high enough concentrations of divalent cations (Ca^2+^ in our case) for proper surface adhesion (Fig. S2†). In samples of pure LPS we found stable spherical structures with a wide size distribution (29 ± 22 nm diameter, measured in AFM images) (Fig. 2A). Mechanical manipulation of the individual LPS vesicles with AFM tip yielded complex, multi-step force spectra (Fig. 2A inset, red curve). During the approach of the AFM tip, at distances exceeding 30 nm, force begins to rise gently. This first step corresponds to vesicular compression which results in the mechanical collapse of the LPS vesicle. In the second step which begins at a distance of 12 nm (which corresponds approximately to the thickness of two bilayers) force rises rapidly. The second step corresponds to the simultaneous compression of the top and bottom layers of the LPS vesicle which by now form a double bilayer. Finally, at an approximate force of 6 nN, the double bilayer becomes mechanically ruptured. The multi-step force spectrum indicates that in the presence of divalent cations LPS forms unilamellar vesicles. Considering that the force required to rupture a pure LPS vesicle (∼6 nN) far exceeds that for pure DMPC (<2 nN), it is predicted that the addition of LPS to DMPC leads to increased membrane stability. Upon heating the LPS vesicles up to 30 °C there were no visible changes in the shapes or sizes of the vesicles (data not shown). Further increase of the temperature (to 50 °C) caused more and more LPS vesicles to melt (*i.e.*, to collapse into a partially flattened patch), but complete melting could not be achieved in a timespan of a few hours (Fig. 2B). This melting might be caused by surpassing the phase-transition temperature of LPS (36 °C). After cooling the sample down to 20 °C, the vesicles remained in the collapsed, molten form, retaining a bilayer height that was the same as measured above 50 °C (Fig. 2C). The height of these molten regions is ∼7 nm, which is 2 nm lower than observed previously by Tong *et al.*, but this difference could arise from either the different AFM probe or the different LPS type used.^8^ The drastic change in height (from ∼12 nm down to ∼7 nm) indicates that during surface adsorption and thermal exposure not only a melting transition, but also a structural rearrangement occurred in the wall of the LPS vesicle, in which the initial double-bilayer patch became a single bilayer. Interestingly, force spectroscopy measurements of LPS layers showed no visible mechanical rupture either in the case of the heated (50 °C) or in the cooled-down (20 °C) samples, indicating that the AFM tip penetrated the bilayers with no detectable resistance (Fig. 2B–C insets). The lack of rupture steps might be caused by irreversible melting that resulted in a demolished membrane structure. Interestingly, during repeated ruptures of LPS vesicles at the same location the integrity of vesicles was compromised, and holes started to appear (Fig. S3†). Considering that the height of the pure LPS patch (∼7 nm) exceeds that of DMPC (∼5 nm), it is predicted that the addition of LPS to DMPC leads to an increase in average SLB height, provided that the two components mix homogenously.

#### Incorporated LPS forms rough domains in DMPC membranes

In the next step towards the synthetic bacterial OM, we mixed LPS with DMPC. As shown in Fig. 3B, LPS-DMPC vesicles formed patches on mica surface similarly to pure DMPC vesicles (see Fig. 1A, 3A). The LPS-DMPC patches were heterogeneous in shape, size and surface roughness. Certain patches had smooth surfaces, whereas others had large, confluent rough parts (Fig. 3B). Smooth fluid-phase lipid regions covered 39%, whereas solid-phase DMPC mixed with LPS formed 61% of the total lipid-covered surface area (Fig. 3B), similarly to the surface coverage ratios of DMPC SLBs (36% fluid, 64% solid, Fig. 3A). The ratio of rough/smooth regions depended on LPS concentration, indicating that the higher, rough-texture regions are formed of LPS molecules. The surface of SLBs lacking LPS (surface roughness, *R*_q_ = 0.77 nm, Fig. 3A) was smoother than that of LPS-rich SLBs (*R*_q_ = 1.16 nm, Fig. 3B). As we increased the LPS concentration from 0.1% up to 0.5%, the total area of rough regions increased correspondingly (from 11% to 56% of the total lipid coverage) (Fig. S4†). The presence of completely smooth patches among the rough ones indicates that LPS did not incorporate into all the vesicles during hydration and extrusion. Interestingly, and in contrast to our prediction, the addition of LPS resulted in a decrease of height in the fluid-phase membrane regions, the mechanism of which is yet unclear. Possibly, LPS may hinder the vertical phase separation of DMPC due to the binding of divalent cations, thereby altering the phase of DMPC. Thus, two fluid-phase DMPC layers on top of each other may arise, similarly to the lowest regions in Fig. 1A. Imaging the rough regions at high resolution revealed three-armed objects (Fig. 3C, white arrowheads), which might be formed by the oriented condensation of multiple LPS oligosaccharide chains. Occasionally, bumps extending 3–4 nm from the patch surface were seen, some of which formed clusters (Fig. 3C, red arrowhead). These bumps may correspond either to supramolecular LPS structures (as the conical shape of the molecule makes it prone to form curved assemblies) or aggregated protein contaminants of the LPS (Fig. S1†).^26^ Previously, Nomura *et al.* found that LPS builds into neutrally charged phosphatidylcholine (PC) membranes, but it phase-separates and forms presumably inverted cubic structures in negatively charged bilayers.^27^ Our findings thus show that, by using the extrusion method, LPS can be incorporated into a DMPC bilayer where it forms rough domains as well as bump-like aggregates.

#### LPS incorporates into solid-phase membrane regions

Considering that the topographically higher regions of the DMPC SLB patches became rough after LPS addition (Fig. 3B), LPS appears to prefer the solid phase. To test this notion further, we added LPS to DMPC SLBs prepared in the presence of Mg^2+^ ions, in which the solid-phase regions showed a spatial arrangement distinctly different from that in the presence of Ca^2+^ (Fig. 4A and C, S5†). While in the presence of Ca^2+^ the solid-phase was internally localized with the fluid-phase confined to the edge of the patch (Fig. 4A), in Mg^2+^ the exact opposite was observed: the solid-phase was localized on one edge, while the fluid-phase was localised internally on the opposite side of the patch with a narrow solid-phase (Fig. 4C). This serendipitous geometric contrast allowed for testing the mechanism whereby LPS attains its structural arrangement in the bilayer. We observed that the overall geometrical arrangement of solid-*versus* fluid-phase regions in LPS-DMPC SLBs (Fig. 4B and D) was identical to that observed with DMPC SLB. Thus, the internal location of LPS observed in LPS-DMPC SLBs (Fig. 3B) is dictated by the solid-phase preference of LPS rather than by LPS phase separation. The solid-phase preference of LPS in DMPC may be explained by the identical length of their fatty-acid chains (14 carbon atoms) but different phase-transition temperatures. While at 20 °C essentially all the fatty-acid chains of LPS are in their longest all-trans state,^28^ a portion of DMPC is in the fluid phase which spatially separates into thin regions (see Fig. 1). Because part of the LPS fatty-acid chain would extend into the aqueous medium, LPS distribution in the fluid-phase regions of DMPC is energetically unfavorable due to hydrophobicity.

#### LPS-DMPC morphology depends on the preparation method

If LPS was added to DMPC prior to the extrusion step, then it incorporated into the membrane (Fig. 3B). If, however, LPS was added to already existing DMPC patches in a separate, subsequent step (DMPC + LPS, see Methods), then some of the LPS formed independent multilayers on top of the DMPC patches, indicating that LPS incorporation was incomplete (Fig. S6,† black arrowhead). LPS multilayer regions displayed a topographical height of 15.2 ± 0.3 nm (Fig. S7†). The remaining LPS either formed independent vesicles or incorporated into solid-state membrane regions. Post-extrusion addition of LPS to DMPC (“DMPC + LPS”) failed to result in a stable membrane. In order to stably incorporate LPS into the DMPC membranes, it needs to be added in advance, prior to extrusion (“LPS-DMPC”). Therefore, to explore the contribution of LPS to the structural dynamics of the membrane, in the following we used LPS-DMPC samples for SLB preparation.

#### LPS distribution follows DMPC phase transition

Upon increasing the temperature of LPS-DMPC SLBs from 20 °C to 30 °C (above the main-transition temperature of DMPC, but below the phase-transition temperature of LPS Ra; ∼36 °C), as the fluid-phase DMPC expanded, the LPS-containing domains shrank drastically (Fig. 5A and B, yellow ROI).^29^ The height difference between fluid-phase DMPC and LPS regions increased by cca. 1 nm, and the surface of LPS domains became smoother (Fig. 5A and B, height profiles). The higher areas are likely formed by condensed LPS molecules. If the sample was cooled back to 20 °C, the rough LPS-DMPC regions started to reappear, originating from the higher regions (Fig. 5C). The most plausible explanation for this redistribution is the appearance of solid-state lipid regions preferred by LPS. The thermal cycle could be repeated several times with the same result. Interestingly, after cooling the sample, LPS coverage wasn't completely reinstated, which might be due to the slower dispersion of LPS in solid-state DMPC at lower temperatures than its condensation in fluid-phase DMPC. The LPS/protein-bumps appeared to be stable during the cycles, suggesting that their structure differs from those of LPS domains. In images of LPS-DMPC at 20 °C, rough regions were also visible, which are likely to be formed by the oligosaccharide chains of LPS in tilted or horizontal positions on the membrane surface. We suggest that LPS molecules not only prefer solid-state membrane regions, but they also induce phase-transition changes around them so that a shift occurs towards the solid state. Upon warming the sample, LPS induced the formation of denser regions, which are also higher than the LPS-covered regions at lower temperatures. We propose a model according to which warming the sample triggers the condensation of LPS, and the height increase is caused by the vertical (perpendicular to the membrane) alignment of the oligosaccharide chains (Fig. 5B, inset). Upon cooling the sample, the dense LPS regions disappear and the oligosaccharide chains return to their initial, tilted or horizontal state. Our results show that LPS localization and its oligosaccharide-chain orientation are highly sensitive to temperature changes, which may have a significant effect on processes that involve LPS recognition. Different LPS types with longer oligosaccharide chains might form even rougher surfaces, and their thermally-induced height increase may be even more prominent.

#### LPS progressively separates from DMPC membranes with time

To test whether and how the presence of LPS affects the dynamics of liposomal shape and size changes and their aggregation, we followed LPS-DMPC liposomes over an extended time span of 10 days and compared their morphological changes to DMPC liposomes (Fig. S8†). DLS was used to follow the changes in the size distribution of the vesicles. Furthermore, we employed AFM to study the morphology of surface-adhered SLBs formed by the maturing liposomes. During the first three days, there were no visible changes on the surface of the patches (Fig. 6A). LPS was distributed in the solid-phase membrane regions creating rough surfaces as seen before (Fig. 3B). After a few days, the edges of the SLB patches became ruffled, the LPS surface coverage of the patches decreased radically, and the background became covered by smaller vesicles of various sizes (Fig. 6B). The pattern of LPS distribution also changed with time. LPS domains began appearing at the edge of the SLB patches, and eventually LPS separated from the membrane to form vesicle-like supramolecular structures (Fig. 6B and C, white arrowheads). The bumps observed in earlier-stage samples (Fig. 3C, red arrowhead) might be the starting points of LPS vesicle formation. The presence of LPS vesicles is also suggested by the emergence of a peak (*d* ∼40 nm) in the size distribution (Fig. 6D, 10th day), similarly to the higher peak of LPS samples. Our results thus suggest that the incorporation of LPS into DMPC membranes results in an energetically unfavourable condition; therefore, regardless of the preparation method, LPS separates from DMPC with time to form independent vesicles. In the outer leaflet of bacterial membranes, this unfavourable state might be overcome by the presence of proteins as well as membrane-bound cations.

### Bacterial outer membrane extraction and reconstitution

Considering that the bacterial outer membrane is not a simple mixture of lipid and LPS but contains a variety of proteins, we tested the effect of adding freshly purified OM proteins on the structure and mechanics of the model membrane. First, we prepared bacterial OM extracts, which contained OM proteins (Fig. S1†) as well as membrane-protein-associated LPS. Interestingly, these samples contained stable particles that did not form SLBs on substrate surfaces (Fig. S9A†). We added this OM extract to DMPC liposomes to allow proteins to be reinserted into the bilayers and to promote protein-rich SLB formation (Fig. S9B†). These SLBs were similar to DMPC bilayers, with the exception of the appearance of bumps on the surface of the patches, which are likely to be OM proteins. Such samples had very few incorporated membrane proteins (Fig. S9B†). Conceivably, a smaller fraction of LPS-free OM proteins were incorporated into the DMPC membrane, while the larger fraction of LPS-associated OM proteins remained in stable OM particles. Although further incorporation may be enhanced by heating, the procedure would result in protein denaturation.

#### OM proteins can be efficiently incorporated into DMPC bilayers with the help of mild detergents

To reinsert as many constituents of the OM into DMPC as possible, we carried out detergent-mediated reconstitution, which allowed us to capture a more native image of what the bacterial surface might look like in SLB form (see Methods). The bilayers formed by surface-adhered OM-DMPC liposomes couldn't be imaged using the same sample preparation techniques as for LPS-DMPC, because the negatively charged proteoliposomes would not adhere to the mica surface even in the presence of divalent cations. To counteract this problem, we prepared PLL-coated positively-charged supporting surfaces onto which OM-DMPC liposomes adhered successfully without the use of divalent cations (Fig. S10,†7A). OM-DMPC SLBs appeared to be higher (∼6 nm) compared to LPS-DMPC SLBs (∼5 nm) (Fig. S10†). Some larger aggregates were visible on AFM images of OM-DMPC as a cause of incomplete solubilization or reconstitution. The adhesion of OM-DMPC SLBs was still weak, but imaging became possible. On the surface of the SLBs smaller bumps were visible which likely correspond to single protein molecules (Fig. S9B,† yellow arrowheads). This presumption is supported by occasional unfolding events recorded while retracting the AFM tip from the bilayer surface (Fig. 7A, inset, S11†). Another interesting property of these SLBs was their periodically striped surface pattern which we did not observe in any of the other samples (DMPC, LPS-DMPC) (Fig. 7A). Individual stripes were ∼0.4 nm in amplitude and displayed a periodicity of 15 nm. Individual stripes extended across distances exceeding a few micrometers. Similar patterned textures were seen by other groups on the outermost surface layers of Gram-negative bacteria would also suggest that this pattern is formed by 2D crystalline monomolecular protein layers,^30–33^ but the texture pattern of such samples show crystalline structures of proteins, not wavelike surfaces as seen in our measurements. Furthermore, such wavelike structures have already been observed on the surface of dried *E. coli* bacteria, providing further evidence that the OM-DMPC model may be an accurate representation of the bacterial OM.^34,35^ Another plausible explanation is that the OM extract contains conically shaped lipids which might induce small local membrane curvatures called ripple phase. However, if this was the case, then the so-called ripple structures should have been very sensitive to temperature changes, and the overall pattern of the stripes would be different.^36^ The most probable explanation is that the wavelike patterns are formed by interactions between DMPC and saturating levels of LPS, which were not visible on LPS-DMPC samples due to the low LPS content. To test this hypothesis, we added high amounts of pure LPS (10% of the total molar amount) to DMPC liposomes and observed the samples after the same detergent-mediated reconstitution process that is normally applied for membrane protein incorporation, in our case OM-DMPC preparation (such high LPS amounts could not be incorporated into membranes by the extrusion method). These high-LPS-content DMPC membranes showed similar surface texture to that of OM-DMPC, thus proving that the striped pattern is indeed the result of interaction between LPS and DMPC (Fig. 7B). In further support, the periodicity of the striped pattern was the same in both samples (∼15 nm). Conceivably, the stripes emerge as the result of a periodical alternation of “LPS hills” and “DMPC valleys” (Fig. 7B and C, height profiles). This theory is further supported by the observation that no protein unfolding events were present in the force spectroscopy data recorded on these samples, thus proving that the stripes emerge as a result of LPS-DMPC interactions, in the absence of proteins (Fig. S12†). An interesting difference between the surface texture of the two samples is that the nematic domain, which is large and uninterrupted in the high-LPS-DMPC sample (Fig. 7B), is small and interrupted by protein molecules in OM-DMPC (Fig. 7C).

### Nanomechanical properties of DMPC, LPS-DMPC and OM-DMPC bilayers

**Fig. 8:**
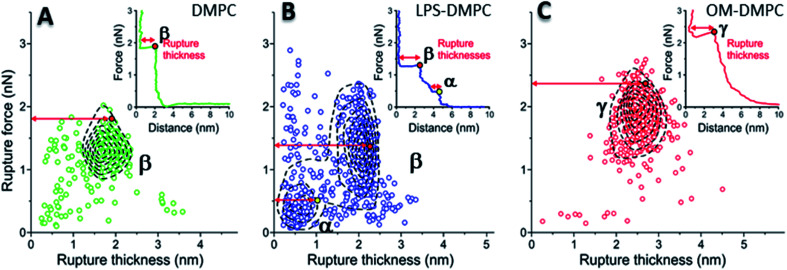
2D maps of the distribution of rupture events of solid-phase DMPC (A), LPS-DMPC (B), OM-DMPC (C) (*n* = 400 for all data sets, but not every point is shown for clarity). Insets show a typical force–distance curve recorded while piercing through the corresponding samples. Red and yellow colored dots explain the difference between “Distance” and “Rupture distance”. Greek letters show populations of breakthrough events. FS measurements were always done at 20 °C.

To reveal the stability of LPS- and OM-protein-containing membranes, we investigated their nanomechanical properties by carrying out force spectroscopy measurements. In these experiments we pierced through the SLBs with the tip of the AFM cantilever until a set level of force was reached. Rupture-force and rupture-thickness distributions are shown in Fig. S13.† Rupture thickness is the distance measured from the point of yield to the subsequent force increase (Fig. S14†). To reveal correlations between rupture force and thickness, we displayed the data in scatter plots (Fig. 8) that contain every membrane-rupture event with force and thickness greater than 0.1 nN and 0.1 nm, respectively. In the scatter plots we were able to identify distinct rupture-event populations. Population α is made up of initial rupture steps characteristic of LPS-DMPC samples, which is followed by population β that is present if a bilayer is ruptured. Population γ emerged in force spectra with single, high-force rupture events, which were characteristic of OM-DMPC samples, and appear to be the result of the combination of α- and β-population events. DMPC bilayers can be ruptured in a single event, which corresponds to the rupture of the bilayer (Fig. 8A, population β). In rare cases, if the membrane was pierced one layer after the other, an additional primary rupture event could be recorded at low force and distance values, which was followed by a secondary event (β) of higher force and lower distance. By evaluating the force–distance curves, we found that rupturing a DMPC bilayer at 20 °C is usually a single-step process, which happens at an average rupture force of 1.3 ± 0.3 nN.^37^ Kaufmann *et al.* have seen that the force started to rise earlier in the case of wild-type LPS-SLBs (wild type in their case was similar to Ra type LPS) compared to pure phospholipid membranes, which implies greater thickness in the presence of LPS,^9^ which is similar to our results. They have measured lower rupture forces in Ra type LPS-SLBs than in palmitoyloleoyl-phosphocholine (POPC). Compared to our results, they have only seen a single rupture event in the force curves, which might be the result of the different type of LPS used. We found that the main difference between DMPC and LPS-DMPC bilayers is not in their average final rupture force, which are essentially identical (1.4 ± 0.5 nN), but in the multistep feature of their force spectra. The average number of rupture steps of LPS-DMPC membranes was two, compared to DMPC membranes which was only one. This extra breaking-through is presumably caused by the presence of the hydrated oligosaccharide-chain layer formed by LPS, which is pierced through prior to bilayer rupture. This difference is also visible if we show all the rupture events of the force curves recorded while piercing through LPS-DMPC bilayers on a map (Fig. 8B). In the case of LPS-DMPC there is a well-defined second cluster of points appearing at the lower force regions compared to DMPC membranes (Fig. 8B, population α), which is comprised by the events of rupturing the hydrated oligosaccharide layer of LPS. α-population events are always followed by a β-population rupture event, whereas β events sometimes (<5%) occur without the preceding α event. This can be explained by the inhomogeneity of the rough LPS-DMPC regions, the tip sometimes penetrated an area within the rough region wherein only DMPC was present. If the DMPC membrane is further reinforced by additional LPS and OM proteins, the thickness of the bilayer increases, and higher force is necessary to pierce through the bilayer (2.03 ± 0.41 nN) (Fig. 8C, population γ). Following a phase of elastic compression, which might be the result of the protein content, rupture usually happens in a single step. We conclude that bilayer stability is greatly increased by the addition of high amounts of LPS and OM proteins, thus our findings suggest that the stability of bacterial OM greatly depends on its molecular constituents and their ratios.

## Conclusion

We succeeded in developing a synthetic bacterial outer-membrane system from DMPC, LPS and OM proteins extracted from *E. coli*. The constituents were mixed step-by-step, and the evoked structural, dynamical and mechanical changes were explored with high-resolution biophysical methods. LPS follows the phase separation of DMPC and is mainly distributed in the solid phase. LPS increased the bilayer height and roughened the bilayer surface. LPS-DMPC bilayers display an additional mechanical rupture step caused by the oligosaccharide layer of LPS. Upon heating, LPS condenses into thicker, solid-phase membrane regions of DMPC, which may play a role in temperature-sensitive lipid raft formation in the bacterial OM, hence molecular recognition processes associated with the cell surface. High LPS and protein content results in a periodically striped surface pattern and increased membrane stability. Preparing SLBs *via* liposome preparation combined with detergent-mediated OM protein and LPS reconstitution thus provides a two-dimensional model of bacterial OM and enables the exploration of the effects of LPS and OM proteins on phospholipid membranes. The synthetic bacterial outer-membrane model provides easier access to investigating the surface binding of various molecules, as well as the infection of bacteriophages.

## Conflicts of interest

There are no conflicts to declare.

## Supplementary Material

NA-003-D0NA00977F-s001
