# Supplementary material for: Development, structure and mechanics of a synthetic E. coli outer membrane model
Source: Nanoscale Adv. 2020 Dec 16;3(3):755–66. doi: 10.1039/d0na00977f (PMC9418885; doi:10.1039/d0na00977f)
Supplement: NA-003-D0NA00977F-s001 [file NA-003-D0NA00977F-s001.pdf]

Electronic Supplementary Material (ESI) for Nanoscale Advances.
This journal is © The Royal Society of Chemistry 2020

Supplementary Information

Development, structure and mechanics of a synthetic E. coli outer membrane model

Bálint Kiss, Tamás Bozó, Dorottya Mudra, Hedvig Tordai, Levente Herényi and Miklós Kellermayer*

Department of Biophysics and Radiation Biology, Semmelweis University, Budapest Hungary

*Corresponding author at: kellermayer.miklos@med.semmelweis-univ.hu

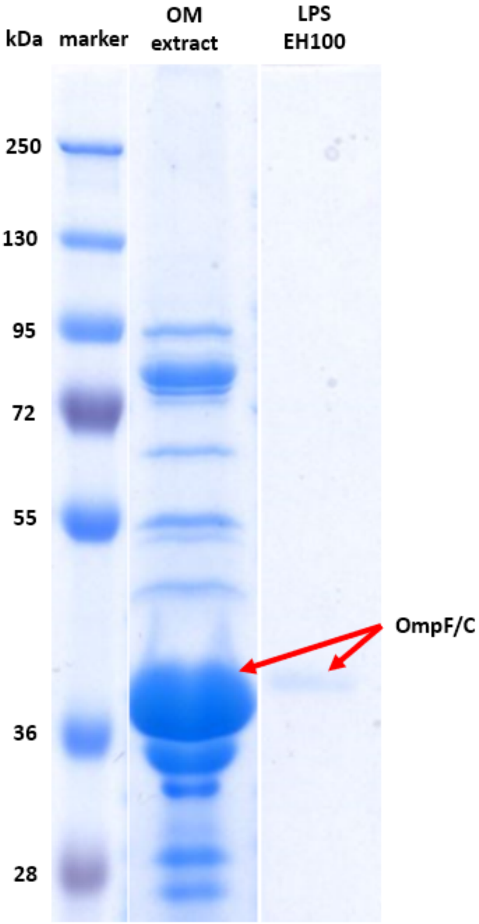

Figure S1. SDS-PAGE results. *E. coli* outer membrane (OM) extracts and lipopolysaccharide samples (LPS EH100) were analyzed by SDS-PAGE, using a 10% acrylamide gel. OM extracts had large quantities of a single type of protein identified, according to molecular weight, as OmpF/C proteins. LPS samples contained traces of a protein of similar size.

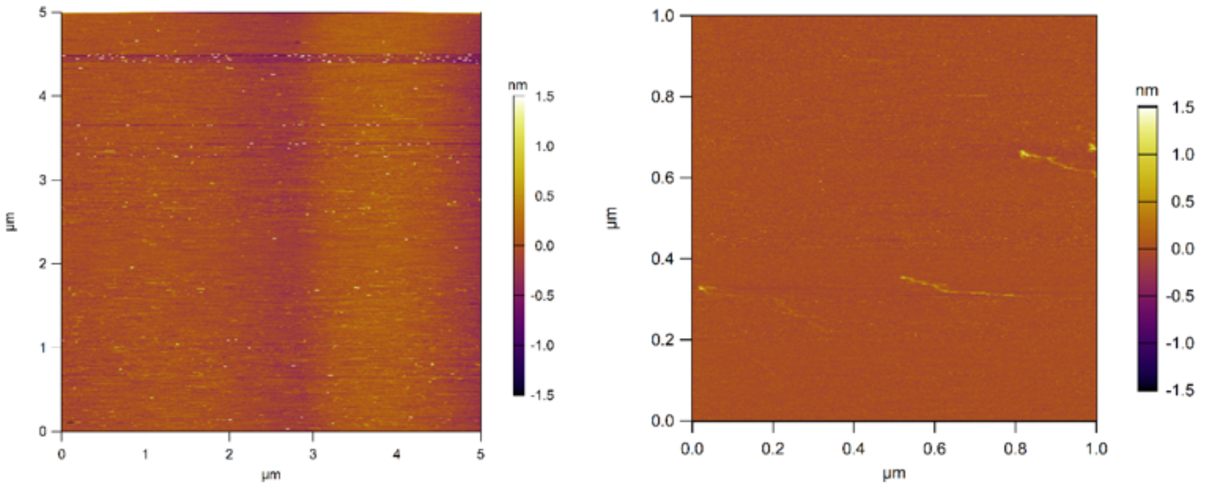

Figure S2. Representative AFM images of the substrate surface collected in two independent experimental attempts carried out to image LPS in the absence of divalent cations.

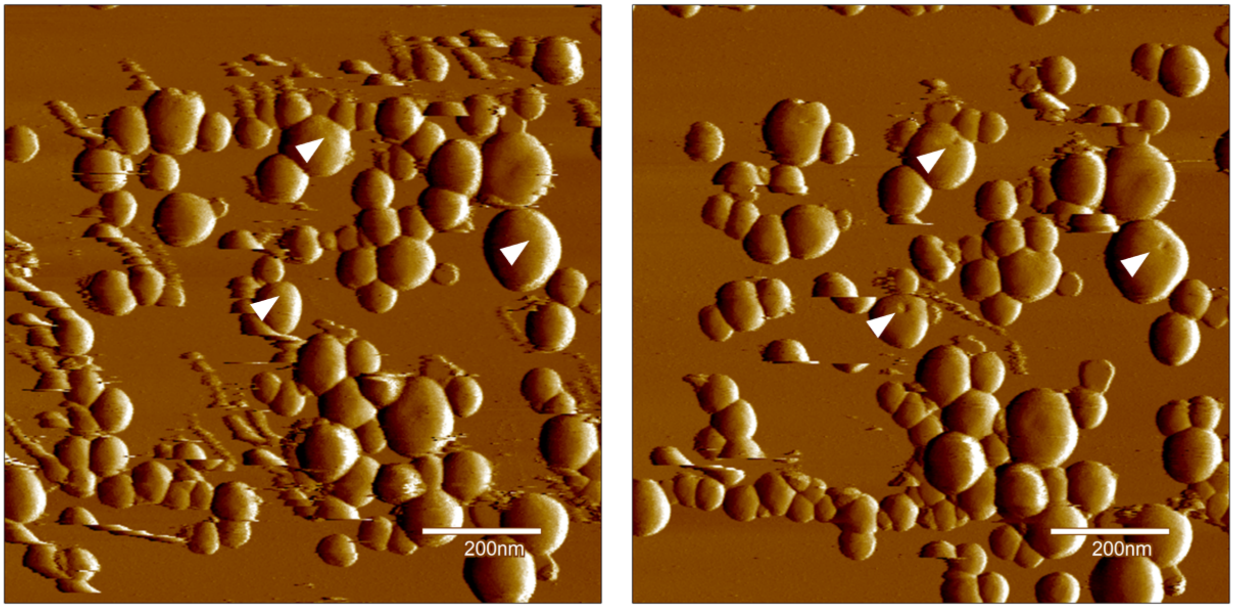

Figure S3. LPS vesicles prior to (left) and following (right) repeated nanoindentations at the locations marked by white arrowheads. Persisting gaping holes were formed.

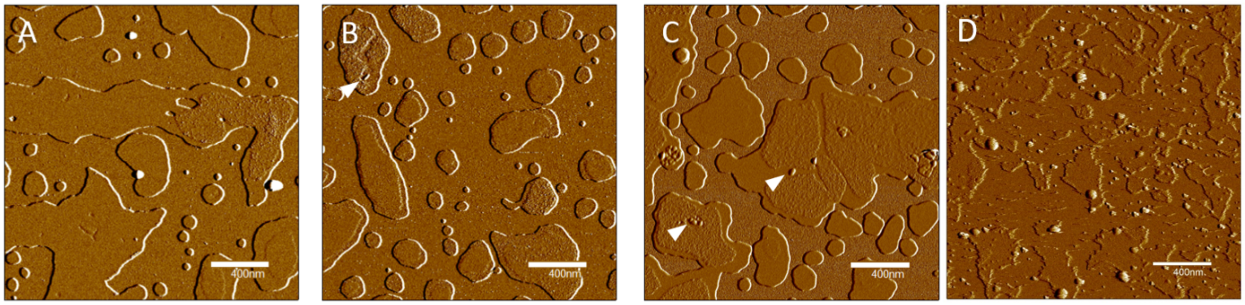

Figure S4. LPS-DMPC with 0.1% (A), 0.2% (B), 0.5% (C), 5.0% (D) LPS molar ratio content. The area of rough membrane regions progressively increases with LPS content.

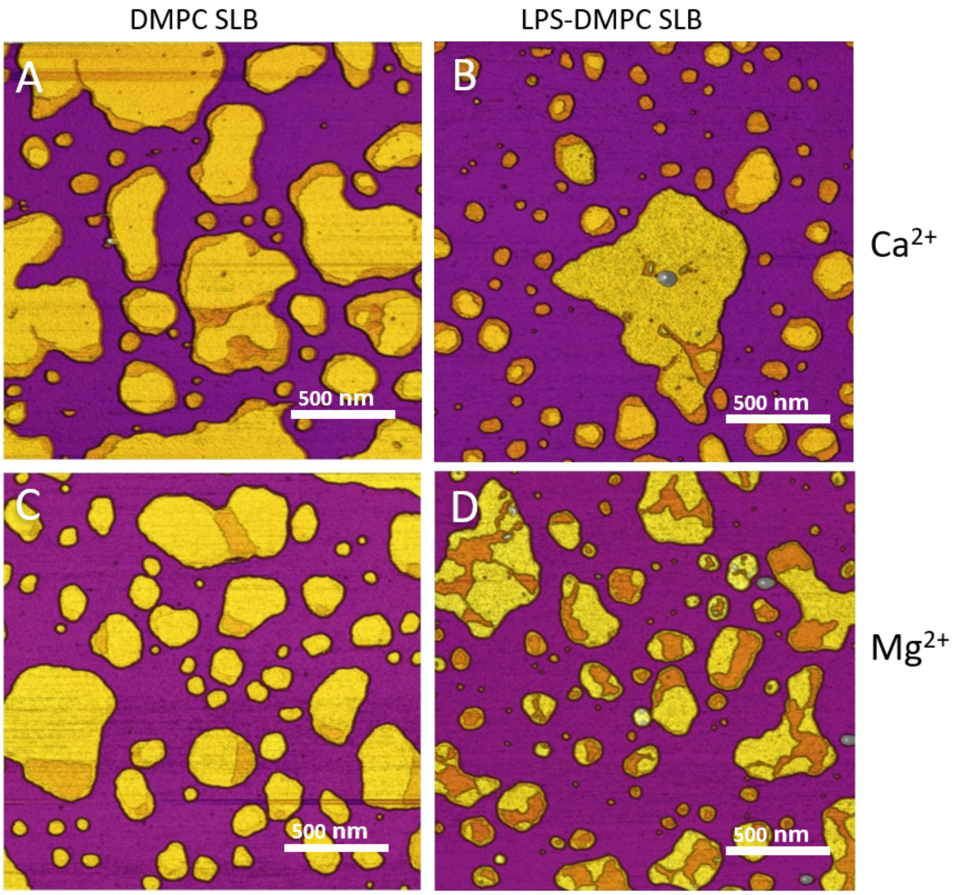

Figure S5. Height-contrast AFM image of DMPC SLB (A and C); LPS-DMPC SLB (B and D); on mica at 20°C in the presence of 10 mM Ca^{2+} (A and B); in the presence of 10 mM Mg^{2+} (C and D) respectively. The spatial pattern of the fluid- *versus* solid-states depends on the cation type. Upon LPS addition, surface roughening occurred in the solid phase regardless of the cation type, indicating that LPS indeed displays a chemical preference for the solid membrane state.

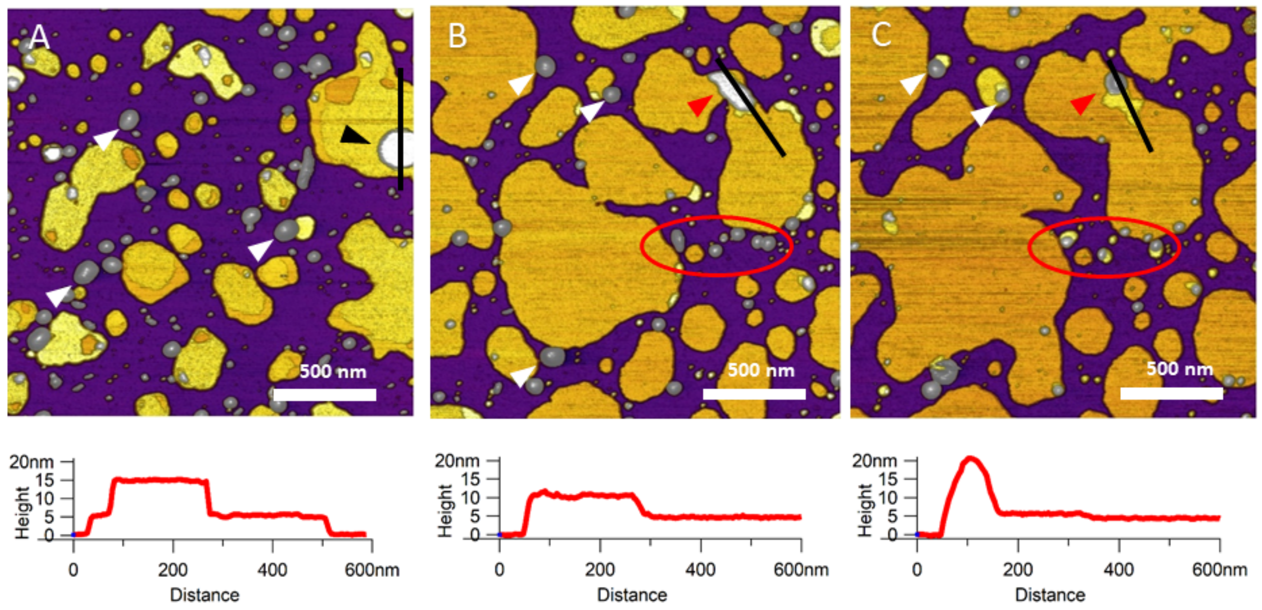

Figure S6. Height-contrast AFM image of DMPC+LPS sample (LPS subsequently added to existing DMPC SLBs, see methods section) in the presence of 10 mM Ca2+ at 20°C (A); and heated up to 25°C (B); then to 40°C (C). B and C were recorded at the same surface location after heating. Black arrowhead shows an LPS multilayer on top of an LPS-DMPC SLB. White arrowheads show LPS vesicles. Red arrowhead shows the location of an LPS vesicle formation. Lower panels show the height-distance functions along the black lines shown in the images. Red ellipse shows a region of interest (ROI) wherein structural changes (melting) of vesicles are visible.

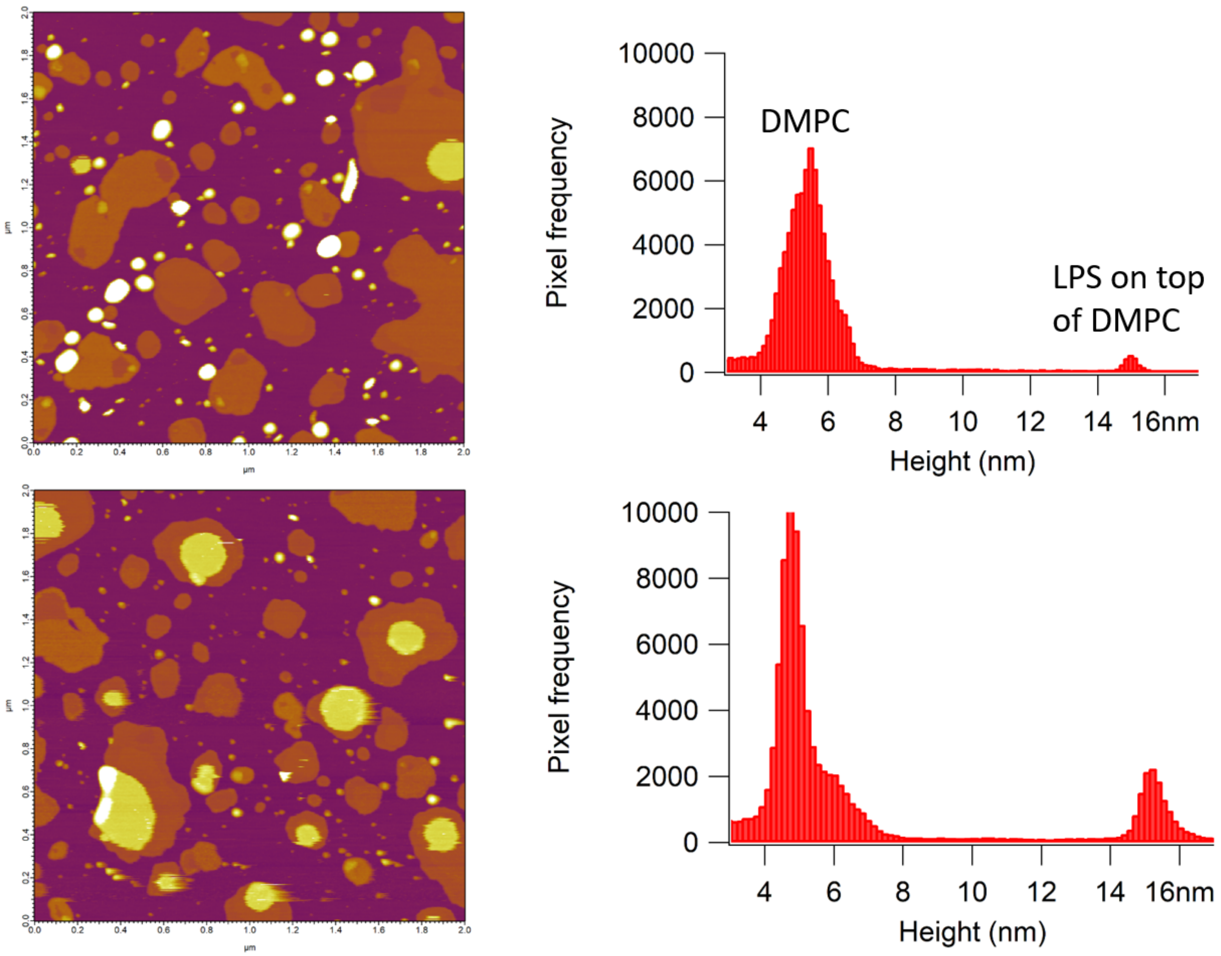

Figure S7. Two representative images of DMPC+LPS samples, as well as their corresponding pixel height distributions on the right. Distributions are bimodal, displaying a second peak of LPS multilayers formed on top of DMPC bilayers.

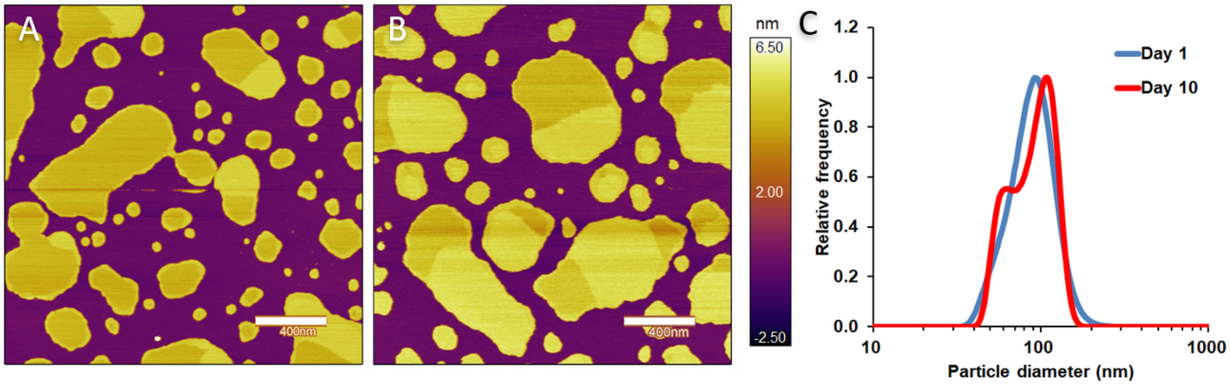

Figure S8. Maturation of DMPC liposomes as a function of time. SLBs formed by DMPC liposomes were imaged on the day of preparation (A), and on the 10th day the stored liposomal sample was pipetted onto mica and imaged again (B). Their size distribution was compared by using DLS (C).

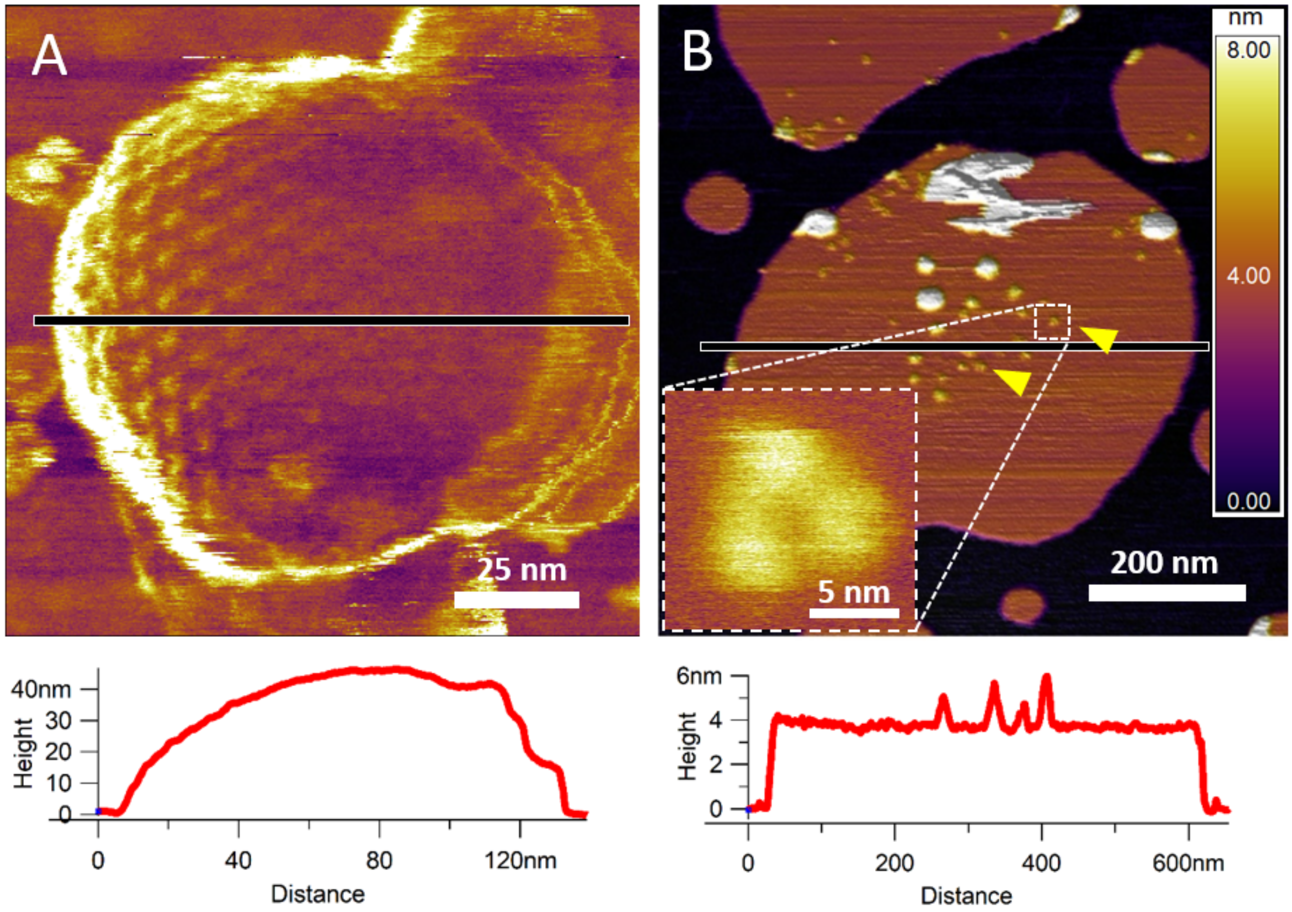

Figure S9. *E. coli* OM extract, passive reconstitution. Phase-contrast AFM image of an OM extract vesicle (A). Height-contrast AFM image of OM-DMPC bilayers. OM proteins were reconstituted into DMPC bilayer without the use of detergents (B). Yellow arrowheads point at single proteins. Inset shows the high-resolution surface structure of a single membrane protein. Lower panels show height profiles taken along the black lines.

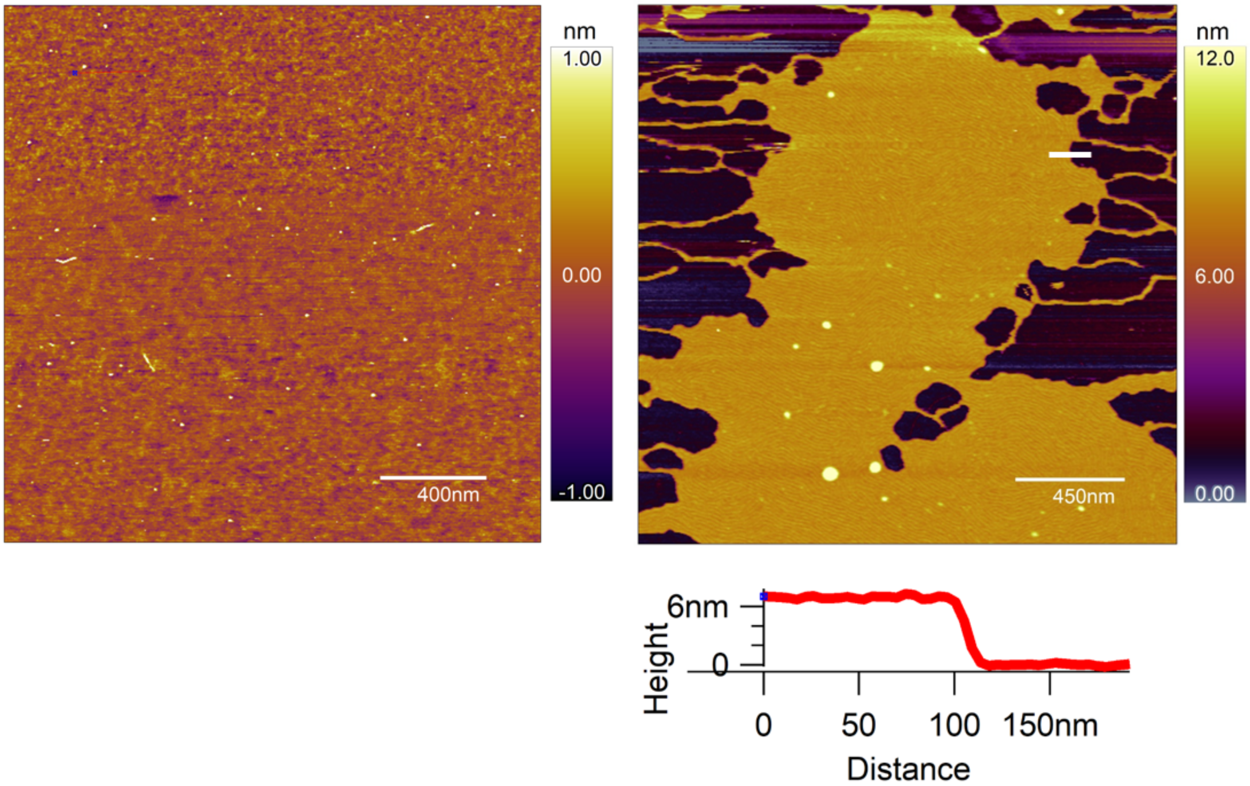

Figure S10. Surface of poly-L-lysine (PLL)-coated mica (left). OM-DMPC SLBs on PLL (right). Cross section of OM-DMPC along the white line (lower right). Both images were recorded at 20 °C

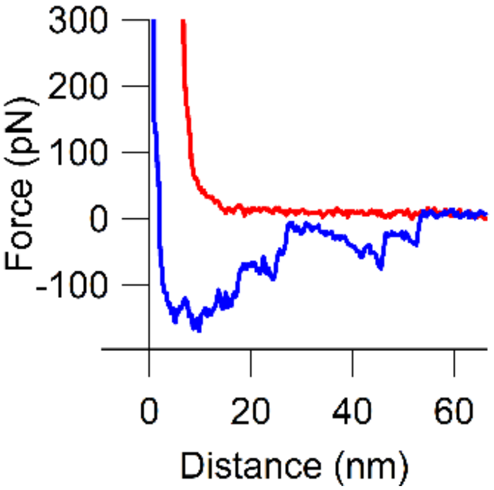

Figure S11. Enlarged view of the force sawteeth in the retraction trace (blue), which correspond to protein unfolding events. This figure is an expansion of the inset of Figure 7A along the force axis.

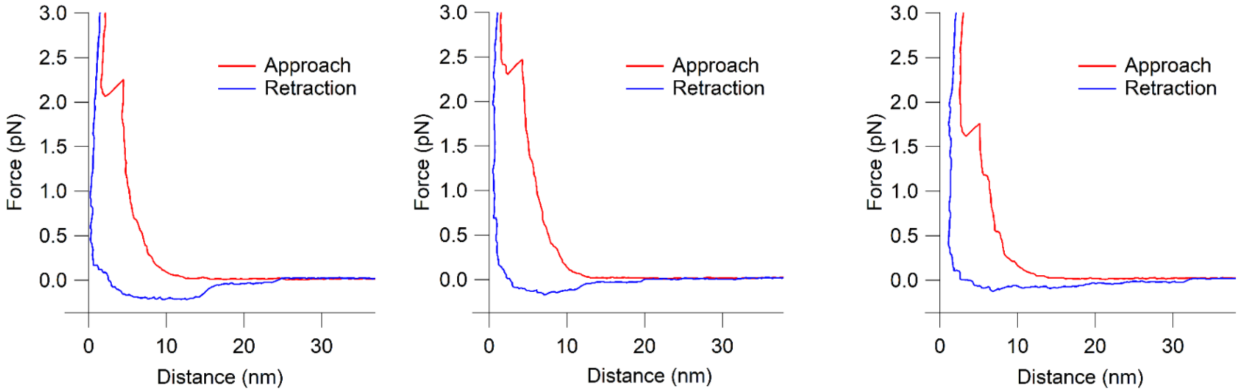

Figure S12. Representative results of force spectroscopy measurements of high-LPS-content-DMPC samples.

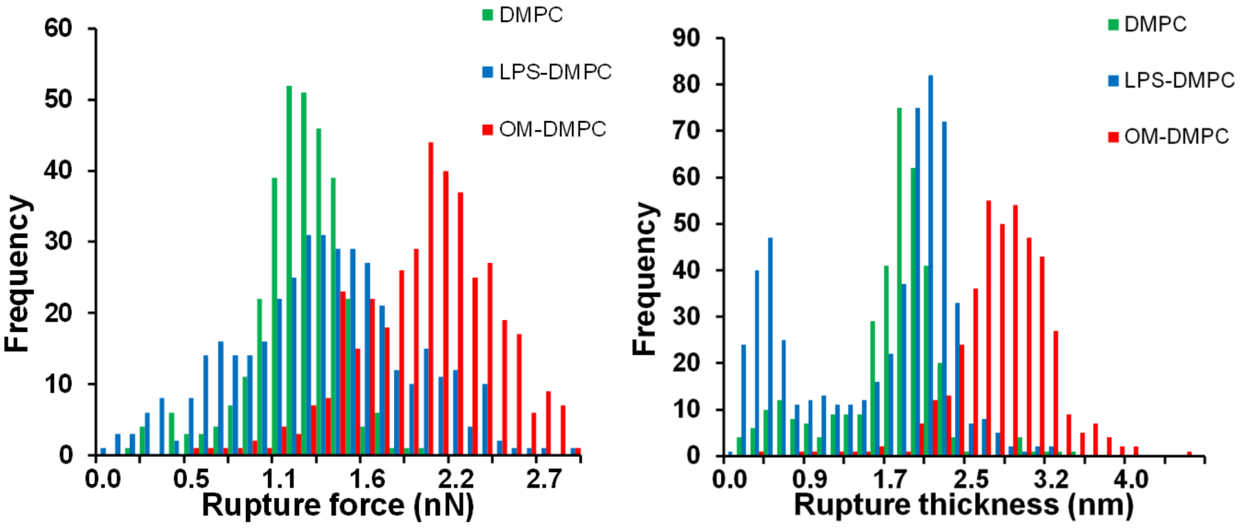

Figure S13. Distribution of rupture force (left) and rupture thickness (right). 400 force curves per sample were analyzed by finding rupture steps with thickness greater than 0.1 nm force greater than 0.1 nN. The results of the analysis are plotted here on a pair of conventional histograms. In Figure 8 of the main text the data were re-plotted in two-dimensional, combined distributions for better visualization and to reveal correlated populations more easily.

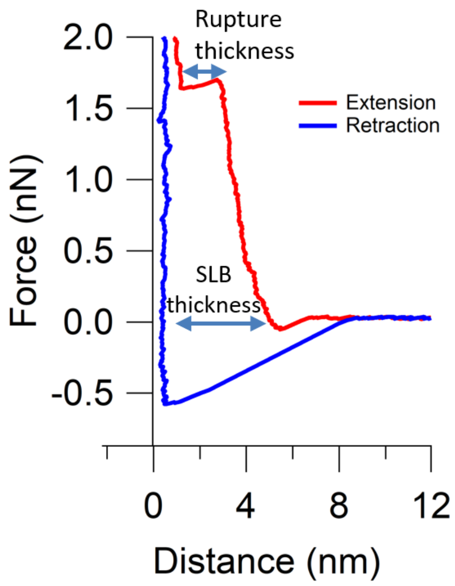

Figure S14. Force spectroscopy recorded while rupturing DMPC bilayer. Annotations explain the difference between rupture and SLB thickness.
